# On Reducing Mortality and Morbidity of Neonatal Surgical Care

**Published:** 2013-01-01

**Authors:** Naeem Khan

**Affiliations:** Department of Pediatric Surgery, KRL Hospital and National Institute of Handicapped Islamabad, Pakistan

Excellent survival of surgical neonates of western countries could not be duplicated in our region. One very important issue is our failure to maintain reproducible and objective records in our institutions. If we were able to do that more accurately, I fear that the figures of high morbidity and mortality will further rise. Here the commentary is not on sporadic and some selected institutions, but to take into account entire health services picture. 

Let me analyze various factors which determine the successes and failures of any service in any country. Monetary constraint is a reality but we should not always take this as an excuse for every difficulty that we face today, after all poverty is also of our own making. Uncontrolled unmonitored multiparity is just one example that keeps us poor and overwhelms us with multitude of premature babies with congenital defects. We have not been able to provide adequate units, appropriately trained medical, nursing and paramedical staff to meet these needs.

Another very important issue is our inability to develop and manufacture highly sophisticated monitoring machines and other equipments which are essential to support the life of a neonate till he becomes independent to sustain his own life. One of the big hurdles here is the maintenance and repair of these equipments which is often a gigantic task. This involves administrative, bureaucratic and financial hurdles. It is often beyond the budget of the hospital in that financial year. Unfortunately patients who need it cannot wait! 

Home deliveries are almost always unmonitored. Consequently, all malformations and defects come to light for the first time only after delivery. These neonates may die because of lack of trained staff, lack of transport, long distances and hostile terrain. For these patients safe transportation and resuscitation is non-existent. Such patients if they reach the hospitals may be overwhelmingly septic, shocked and hypothermic. This is an important factor of high morbidity and mortality. In providing the optimal neonatal care, perhaps, the best option is to form a fully developed neonatal care setup that is easily accessible to obstetric units of every medical institution. 

Scrupulous discipline of neonatal surgical unit by dedicated and vigilant staff plays an important role in the running of such a unit. Here understanding and enforcing asepsis is of paramount importance. Hand washing or hand disinfection using readily sterilizing solutions is part of neonatal care and it needs absolute compliance. Although machines have taken over several aspects of monitoring of a patient, keen observation of respiratory efforts, peripheral perfusion, activity, lethargy, abdominal distension, feed intolerance failure to pass stool and urine measurements are important clinical observations by the attending staff which require urgent corrective measures to ward off irreversible situation. Similarly calculating the fluid requirements and drug dosages are the responsibility of knowledgeable and educated staff, who should also be able to chart observation sheet accurately and be able to communicate any untoward change to the senior staff. 

In neonatal surgery adequate facilities of operating room are of paramount importance. Ideally these involve special equipment and material suited to neonatal surgery such as warming blankets, small-size instruments, fine sutures, fine tubes and neonatal anesthetic instruments Curbing the tendency of individualistic approach is important for improvement. Team work is the real need of the day. In present times of limited resources, scarcity of trained staff and high costs of health care, we have found a combined neonatal intensive care unit, wherein neonatologists, physicians, radiologists, pathologists and surgeons work with a team sprit, is more productive. In this model, frank discussions provide better information and help in improving the patient care. 

From the foregoing account it is abundantly clear that we have not yet made sufficient efforts to provide adequate services to our surgical neonates. Till such time, although we may continue to report episodic successes, this by no means can be termed as an organized service with sustained and reproducible results. Here it is also important to note that progress in every walk of life should be parallel, may it be machine manufacturing, transport and communication, hospital facilities, standards of education, and in-service training. Above all no one should get the opportunity to question the dedication and sacrifice of the hospital staff. Finally we have a long way to go in achieving the goal of family planning, patient education and health insurance for all.

## Footnotes

**Source of Support:** Nil

**Conflict of Interest:** None

